# Montelukast Disposition: No Indication of Transporter-Mediated Uptake in OATP2B1 and OATP1B1 Expressing HEK293 Cells

**DOI:** 10.3390/pharmaceutics7040554

**Published:** 2015-12-15

**Authors:** Marie Brännström, Pär Nordell, Britta Bonn, Andrew M. Davis, Anna-Pia Palmgren, Constanze Hilgendorf, Katarina Rubin, Ken Grime

**Affiliations:** 1 Respiratory, Inflammation and Autoimmunity Innovative Medicine, AstraZeneca R&D Gothenburg, 431 83 Mölndal, Sweden; E-Mails: britta.bonn@astrazeneca.com (B.B.); andy.davis@astrazeneca.com (A.M.D.); anna-pia.palmgren@astrazeneca.com (A.-P.P.); katarina.rubin@astrazeneca.com (K.R.); ken.grime@astrazeneca.com (K.G.); 2 Drug Safety and Metabolism, AstraZeneca R&D Gothenburg, 431 83 Mölndal, Sweden; E-Mails: par.nordell@astrazeneca.com (P.N.); constanze.hilgendorf@astrazeneca.com (C.H.)

**Keywords:** montelukast, OATP2B1, OATP1B1, hepatic uptake, transporters

## Abstract

Clinical studies with montelukast show variability in effect and polymorphic OATP2B1-dependent absorption has previously been implicated as a possible cause. This claim has been challenged with conflicting data and here we used OATP2B1-transfected HEK293 cells to clarify the mechanisms involved. For montelukast, no significant difference in cell uptake between HEK-OATP2B1 and empty vector cell lines was observed at pH 6.5 or pH 7.4, and no concentration-dependent uptake was detected. Montelukast is a carboxylic acid, a relatively potent inhibitor of OATP1B1, OATP1B3, and OATP2B1, and has previously been postulated to be actively transported into human hepatocytes. Using OATP1B1-transfected HEK293 cells and primary human hepatocytes in the presence of OATP inhibitors we demonstrate for the first time that active OATP-dependent transport is unlikely to play a significant role in the human disposition of montelukast.

## 1. Introduction

Montelukast, a leukotriene receptor (cysLT1) antagonist, is used for the treatment of asthma. In mono-therapy it is used as an alternative to low-dose inhaled corticosteroids (ICS) or in addition to ICS for improving clinical management of the disease in various phenotypes of asthma patients [1]. The heterogeneity of asthma is receiving increasing attention in order to better customize treatment according to different clinical and biological phenotypes. Montelukast, itself, is subject to high inter-individual variability in response which limits its effectiveness [2]. In contrast, it is noteworthy that following 10 mg oral administration of montelukast to healthy volunteers, the reported oral bioavailability is in the region of 60% with low variability and low clearance of approximately 2% liver blood flow, again with low variability [3,4]. Despite this, Organic Anion Transporter Protein 2B1 (OATP2B1)-dependent human intestinal absorption (specifically the single nucleotide polymorphism (SNP) SLCO2B1*3) has been suggested as a possible cause for observed patient variability in effect [5]. OATP2B1 is an uptake transporter located in the luminal membranes of human small intestinal enterocytes, in the sinusoidal membrane of hepatocytes, mammary glands, placenta, heart, and skeletal muscle [6]. The claim relating variability in montelukast effect to OATP2B1 expression cited above has been disputed [7]. Since it is important to understand if this is a point of differentiation for novel drug discovery projects which aim to provide more comprehensive allergic asthma therapy, we performed experiments in an attempt to provide further clarity.

## 2. Experimental Section

### 2.1. Materials

[^3^H]-Montelukast was supplied by The American Radiolabeled Chemical Company (St. Louis, MO, USA) and non-radiolabeled montelukast was supplied by Chemtronica (Stockholm, Sweden). Montelukast is light sensitive and was stored in darkness. [^3^H]-Estrone 3-sulfate (E_1_3S) and [^3^H]-estradiol 17-beta-glucuronide (E_2_17βG) were obtained from PerkinElmer (Waltham, MA, USA), whilst non-radiolabeled E_1_3S, E_2_17βG, rifampicin, dimethyl sulfoxide (DMSO), 2-(*N*-morpholino)ethanesulfonic acid (MES), and fetal calf serum (FCS) were obtained from Sigma–Aldrich (St. Louis, MO, USA). Erlotinib was obtained from Chemtronica (Stockholm, Sweden); all cell culture reagents, Leibovitz medium supplemented with l-glutamine, Hank’s balanced salt solution (HBSS), phosphate buffered saline (PBS), and HEPES from Invitrogen Life Technologies (Paisley, UK); sodium hydroxide (NaOH) and hydrochloric acid (HCl) from Merck KgaA (Darmstadt, Germany), and the scintillation cocktail Optiphase “Hisafe” 2 was provided by PerkinElmer (Waltham, MA, USA). Pierce^®^ BCA Protein Assay kit was used for protein determination (Thermo Scientific, Waltham, MA, USA).

### 2.2. Cell Culture

Stable HEK293 cell lines, either expressing human OATP2B1/SLCO2B1 (HEK-OATP2B1) or transfected with empty vector (HEK-mock) were produced at the University of Uppsala, Sweden, as detailed previously [8]. OATP1B1/SLCO1B1 (HEK-OATP1B1) and corresponding mock transfected HEK293 cells were produced in-house at AstraZeneca [9]. The cells were thawed and cultured in 25 cm^2^ flasks (Nunc, Thermo Scientific, Waltham, MA, USA) at 37 °C in an atmosphere of 95% air and 5% CO_2_ using antibiotic free cell culture medium for the first passage: Dulbecco’s Modified Eagle Medium (DMEM) with Glutamax (500 mL) containing heat inactivated FCS (50 mL). In the continuous culture hygromycin B (75 µg/mL) was used for the HEK-OATP2B1 and 1% G418 for the HEK-OATP1B1 culture. In the OATP2B1 uptake studies, the cells were seeded in CellBIND 24-well plates (Corning Costar^®^ Corporation, Corning, NY, USA) at a density of 600,000 cells/well and experiments were conducted two days after seeding. HEK-OATP2B1 and HEK mock cells were used at passages 4 to 10 and passages 15 to 22, respectively. The OATP1B1 uptake studies were performed two days following cell seeding (density of 250,000 cells/well) in poly-lysine-d-coated 24-well plates (BD Biosciences, San José, CA, USA) with the cells used at passages 33 to 40 (HEK-OATP1B1) and passages 13 to 20 (HEK-mock cells).

### 2.3. HEK-OATP Uptake Experiments

Montelukast, E_1_3S, and E_2_17βG dose solutions were prepared as a mixture of [^3^H] compound (1 µCi/mL) and non-radiolabeled compound. Uptake experiments were performed in a thermostated shaker, set to 37 °C and 250 rpm (Thermostat; BMG Lab Vision, Rotenberg, Germany). At the start of the experiment, the cell culture medium was removed and the cells were washed twice with pre-heated transport media (700 µL per well). Thereafter, the cells were incubated in pre-warmed transport media (200 µL) for 10 min and experiments initiated by the addition of an equal volume of pre-warmed dose solution (200 μL) to each well. All uptake experiments were carried out in transport medium consisting of HBSS buffered with 25 mM MES adjusted to pH 6.5 with NaOH or buffered with 25 mM HEPES adjusted to pH 7.4 with NaOH. All experiments were performed in triplicate and the total concentration of DMSO and ethanol never exceeded 1% and 0.2% (*v*/*v*), respectively, in the incubations.

For the OATP2B1 time-dependent experiments, E_1_3S (0.5 µM) and montelukast (1 µM) were incubated at pH 6.5 and 7.4 from 0.5 to 20 min. Concentration-dependent and transport inhibition experiments were performed for 3 min at pH 6.5, with E_1_3S (0.02 to 50 μM) and montelukast (0.02 to 6.6 μM). E_2_17βG (1 µM) and montelukast (1 µM) were incubated at pH 7.4 from 0.5 to 20 min with HEK-OATP1B1 and corresponding mock transfected cells. Concentration-dependent experiments were performed for 1 min with E_2_17βG (0.02 to 50 µM).

The uptake experiments were terminated by removing the incubation media and washing the cells twice with PBS. Ice-cold NaOH (0.2 M, 500 μL) was added to each well and the plates were placed in a refrigerator for at least one hour to lyse the cells. The cell homogenate was neutralized with an equal volume of HCl (0.2 M) prior to sampling for protein determination and scintillation counting for radioactivity analysis. The absorbance was read at 562 nm using a SpectraMax 190 plate reader (Molecular Devices, Sunnyvale CA, USA). The samples were assayed for radioactivity using a Wallac WinSpectral™ 1414 liquid scintillation counter and a Tri-Carb 3100TR Liquid Scintillation analyzer, both supplied by PerkinElmer (Waltham, MA, USA). Scintillation cocktail Optiphase “Hisafe” 2 was added to each sample before analysis.

*K*_m_ and *V*_max_ for OATP2B1 and OATP1B1 substrates were obtained by fitting to a Michaelis–Menten equation (GraphPad Prism, Version 6.01, GraphPad Software Inc., San Diego, CA, USA). OATP-mediated uptake, calculated by subtracting the uptake in HEK-mock from that in HEK-OATP cells was used for the Michaelis–Menten fit.

The enterocyte blood flow method [10] was used to calculate the intestinal drug concentration experienced by the drug transporter after a 10 mg dose (drug concentration = fraction absorbed × absorption rate constant × dose / enterocyte blood flow of 18L/h).

### 2.4. Human Hepatocyte Uptake Experiments

Pooled cryopreserved primary human hepatocytes were obtained from Bioreclamation IVT (10-donor batch IRK, Brussels, Belgium). Hepatocyte uptake was assessed using an “oil-spin” procedure, as previously described [11]. Incubations with montelukast or E_1_3S (1 µM) were performed in serum free Leibovitz L-15 medium in absence and presence of OATP transport inhibitors rifampicin (10 µM) or erlotinib (4 µM) at a final hepatocyte concentration of 1,000,000 cells/mL. Briefly, cells were thawed and re-suspended in Leibovitz medium according to the manufacturer’s protocol, counted using a CASY cell counter (Innovatis AG, Bielefeld, Germany) and further diluted to reach a concentration three times that of the final incubation. Incubations were initiated as follows: substrate and inhibitor solutions were prepared in Leibovitz medium to a concentration three times that of the final incubation. A blank (no inhibitor) solution was also prepared with the same DMSO concentration as the inhibitor solutions. Equal volumes (300 µL) of blank/inhibitor solution and cell suspension were mixed in a glass vial and allowed to pre-incubate for 15 min in water bath set to 100 rpm (linear shaking) at 37 °C. Experiments were started by addition of substrate solution (300 µL). The final DMSO content in all incubations was 0.15% *v*/*v*. At designated time points (15, 30, 45, 60, 120, and 180 s) a 100 µL aliquot of incubate was removed and dispensed into a microtube containing an upper layer of oil and a lower layer of cesium chloride. Upon rapid centrifugation (7000 *g*; 15 s) hepatocytes are passed through the oil layer separating them from the incubation medium. The microtubes were directly placed on dry ice before cutting (just below the oil/cesium chloride interface) of the frozen tube tips containing the cell pellets into a 96-well plate for sample processing as previously described [11]. Incubations were performed in triplicate.

Samples were analyzed by LC–MS–MS: HTS PAL injector (CTC Analytics, Zwingen, Switzerland), HP 1100 LC binary pump (Agilent Technology, Waldbronn, Germany) and a Micromass Quattro Ultima mass spectrometer (Waters Corp., Milford, MA, USA). A mobile phase (A: 0.2% formic acid, 2% acetonitrile; B: 0.2% formic acid in acetonitrile) with a flow rate of 0.7 mL/min was used with a Sunniest C18 RP-aqua column (ChromaNik Technologies Inc, Osaka, Japan) for chromatography.

Relative rates of uptake were determined by linear regression. Data generated up to 60 s after substrate addition gave for montelukast as well as for E_1_3S profiles the optimal statistical confidence in slope determination and was, therefore, included in analysis. Significance of inhibition of uptake by rifampicin and erlotinib was calculated from a two-tailed linear regression *t*-test (in-built functionality Prism 5, GraphPad Software, USA). Obtained *p*-values were corrected for multiple comparisons using the Holm–Bonferroni method [12]. Adjusted *p*-values < 0.05 were considered as significant.

## 3. Results and Discussion

### 3.1. HEK-OATP Uptake Experiments

**Figure 1:**
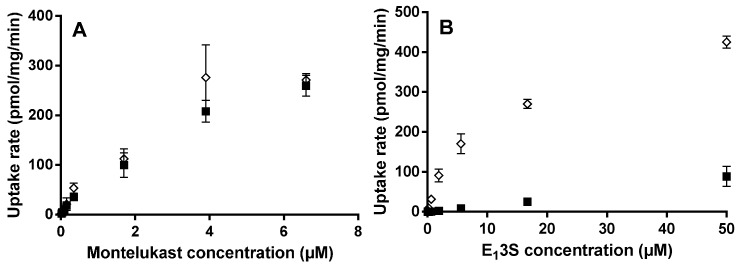
Concentration-dependent uptake of montelukast (**A**) and E_1_3S (**B**) in HEK-OATP2B1 (◊) and HEK-mock (■) at pH 6.5. Results are given as mean ± S.D., *n* = 3.

At pH 6.5 using 3 min incubations, eight concentrations (0.02–6.6 μM) of montelukast failed to show a difference in cell uptake rate between the HEK-mock and the HEK-OATP2B1 cells (Figure 1A). The solubility of montelukast limited the upper concentration to 6.6 μM. However, it is likely that the intestinal concentration after an oral dose will be similarly limited. Using the enterocyte blood flow method [10] to calculate the intestinal drug concentration experienced by the drug transporter after a 10 mg dose, the estimated relevant concentration of montelukast to study, is equal to 0.6 μM. Eight concentrations of E_1_3S (0.02–50 μM), also incubated for 3 min at pH 6.5 in HEK-OATP2B1 and HEK-mock (Figure 1B), were used for defining *K*_m_ and *V*_max_ values of 7.5 μM and 377 pmol/mg protein/min for the incubations. The OATP2B1-mediated uptake for E_1_3S, was used for the Michaelis–Menten fit in order to obtain the *K*_m_ and *V*_max_. (The time-dependent uptake for montelukast and E_1_3S at pH 6.5 and the Michaelis–Menten fit is provided in Figures S1 and S2, respectively).

**Figure 2:**
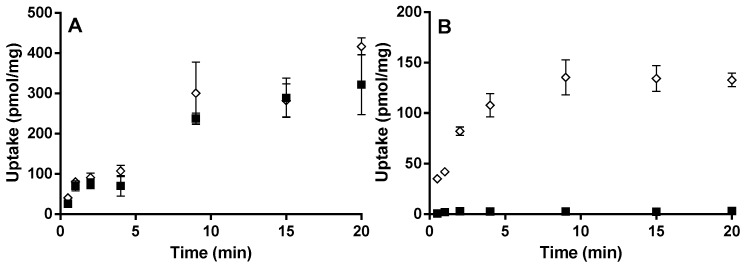
Time-dependent uptake of (**A**) 1 µM montelukast and (**B**) 0.5 µM E_1_3S at pH 7.4 in HEK-OATP2B1 (◊), and HEK-mock cells (■). Results are given as mean ± S.D., *n* = 3.

Since a high passive uptake could mask a lower active uptake, the incubation pH was increased to 7.4, less favorable for the passive transport of montelukast due to increased ionization of the carboxylic acid. Only single concentrations of E_1_3S (0.5 µM) and montelukast (1 µM) were used. At pH 7.4, the uptake of montelukast observed in both HEK-OATP2B1 and HEK-mock cells was similar (Figure 2A). E_1_3S showed higher uptake in HEK-OATP2B1 transfected cells than in HEK-mock cells (Figure 2B), indicating that the assay was able to detect substrates of OATP2B1 also at pH 7.4.

**Figure 3:**
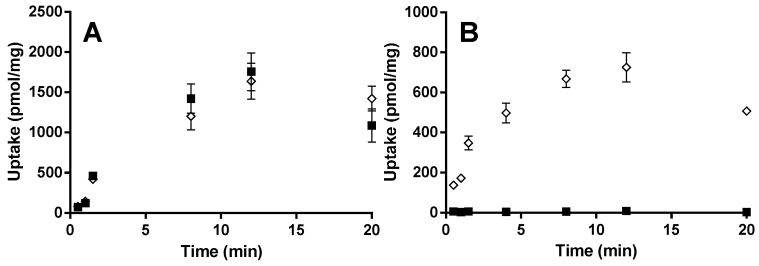
Time-dependent uptake of (**A**) 1 µM montelukast and (**B**) 1 µM E_2_17βG in HEK-OATP1B1 (◊) and HEK-mock cells (■) at pH 7.4. Results are given as mean ± S.D., *n* = 3.

In the time-dependent uptake experiment of montelukast (1 µM) with HEK-OATP1B1 and HEK-mock cells performed at pH 7.4, no difference in uptake rate was detected between the two cell lines (Figure 3A). E_2_17βG (1 µM) was used as a positive control for validation of the OATP1B1-mediated uptake and the uptake rate was notably higher in HEK-OATP1B1 than in HEK-Mock cells (Figure 3B). *K*_m_ and *V*_max_ values were estimated to be 4.1 µM and 697 pmol/mg/min, respectively, using Michaelis–Menten kinetics (Figure S3). The OATP1B1-mediated uptake, was used for the Michaelis–Menten fit.

### 3.2. Human Hepatocyte Uptake Experiments

**Figure 4:**
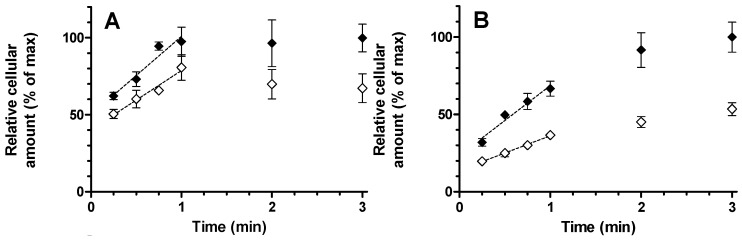
Time-dependent uptake of (**A**) montelukast and (**B**) E_1_3S into human hepatocytes. Incubations were performed with substrate alone (♦) and in the presence of 10 µM rifampicin (◊). Relative initial uptake rates were assessed from linear fit to data obtained from 15 to 60 s (dashed). Results are given as mean ± S.D., *n* = 3.

Both montelukast and E_1_3S were taken up into human hepatocytes linearly with respect to time between 15 and 60 s, when incubated at 1 μM. Rifampicin-mediated (10 μM) statistically significant 51% inhibition of E_1_3S uptake (adjusted *p* < 0.001) but no significant impact on montelukast uptake (adjusted *p* = 0.16) (Figure 4). Erlotinib (4 μM) did not significantly change the rate of uptake for either E_1_3S or montelukast (adjusted *p* = 0.8 and 0.56, respectively, Figure S4).

## 4. Conclusions

Previously, it has been reported that OATP2B1 may be important in the human intestinal absorption of montelukast [5]. These conclusions were drawn from *in vitro* Caco-2 data in which the relationship between permeation rate and montelukast concentration was interpreted as being indicative of transporter mediated transport that could be described by Michaelis–Menten kinetics. Only the single concentration of 170 µM montelukast deviated from the linear relationship between rate and concentration, this data point being associated with the greatest variance, possibly due to limitations in the aqueous solubility of montelukast being in the 10–50 µM range [13]. Vectorial transport of montelukast through MDCKII cells transfected with OATP2B1 was shown by the authors to be higher (1.4-fold) compared to control MDCKII cells. Although the result was calculated to be significant, the assay had a relatively small “window” even for the positive control E_1_3S (only 2.8-fold higher cell uptake in OATP2B1 cells compared to control). Additionally, the cell uptake was shown to be inhibited 27% by 125 µM sulfobromophthalein. In this context it is noteworthy that when E_1_3S has been previously used as a substrate of OATP2B1, an IC_50_ of 2 µM was reported for sulfobromophthalein [14]. Other investigators have subsequently challenged the conclusions of Mougey and co-workers [7]. Chu *et al.* provided MDCKII (control and OATP2B1-expressing) cellular uptake data that showed no significant difference in the amount of montelukast taken into control and OATP2B1 cells when incubated at 1 µM, pH 6.0. The same finding was observed at incubation pH 7.4, but in both cell lines the amount of drug taken up was approximately 1.5-fold lower compared to incubations performed at pH 6.0. A similar experiment was performed using 1 µM E_1_3S and the rate of uptake was approximately 10-fold higher in the OATP2B1 cells compared to control cells. Thus, an assay with a relatively large window for the positive control compound contradicted the findings of the earlier publication. Furthermore, cellular accumulation data for 1 µM E_1_3S and 3 µM montelukast presented by Chu *et al.*, concluded that E_1_3S accumulated to a greater extent in MDCKII OATP2B1 cells when added to the basolateral side than the apical side, but that this was not true for montelukast. However, higher montelukast accumulation in the control cells after apical administration, compared to basolateral administration, together with only a two-fold difference between basolateral and apical administration for E_1_3S, reduces the clarity of these results.

Here we performed experiments in an attempt to describe montelukast time- and concentration-dependence for HEK293 OATP2B1 cellular uptake. A novel human OATP2B1 expressing HEK293 cell line was used, created at Uppsala University [8]. This cell line used was validated with the OATP substrate E_1_3S, shown to have a *K*_m_ value of 7.5 µM, in excellent agreement with previously observed values [15,16]. Drug transport was measured using HEK-OATP2B1 and HEK-mock (empty vector) cells at pH 6.5, consistent with the luminal pH of the small intestine and also because the transporter is reported to have a higher substrate transport capacity at acidic pH [17]. Since high passive uptake could potentially still mask an active component, the experiment was repeated at 1 µM and the higher pH of 7.4, in order to reduce the passive permeability through decreasing the percentage of the un-ionized drug. Large differences in E_1_3S uptake were observed when comparing the OATP2B1- and mock-transfected HEK cell lines at both pH 6.5 and 7.4. This gave an advantageous window for defining test compounds as substrates of the drug transporter. In contrast to E_1_3S, montelukast uptake at pH 6.5 was very similar in the OATP2B1 and mock cell lines over a range of concentrations. Altering the pH in the incubation from 6.5 to 7.4 did not affect the OATP2B1/mock ratio substantially for E_1_3S. We, therefore, conclude that montelukast is not pronouncedly transported by OATP2B1 due to high inherent passive permeability and given the low variability in observed human exposure there is no reason to consider that the transporter is important in driving human intestinal absorption [3,18]. This data therefore supports a recent clinical study indicating that OATP2B1 polymorphisms do not affect the pharmacokinetics of montelukast [19].

It is interesting to note that montelukast has been shown to be a relatively potent inhibitor of OATP1B1, 1B3, and 2B1 [20,21]. It is also worthy of note that the drug has been postulated to be actively transported into human hepatocytes *in vitro*, although the mechanism of the uptake has, to our knowledge, never been investigated [22]. Drugs that interact with OATP1B1 are typically acids and are most likely to be moderately to highly lipophilic with log*P* values in the range 3.5 to 5.5 [23]. Montelukast is more lipophilic than this, having a log*P* of over 7. Whilst acidic drugs that are highly bound to plasma proteins typically have low steady state volume of distribution (*V*_ss_) values in the order of 0.1 to 0.3 L/kg, for those whose hepatic disposition is influenced by OATP1B1/3 transport, the *V*_ss_ is likely to be larger [24,25]. Montelukast does not obviously fit as having OATP1B1 driven disposition, having a *V*_ss_ of approximately 0.15 L/kg [4]. In the studies described in this manuscript we used HEK293 OATP1B1 and the corresponding HEK-mock (empty vector) cells in an attempt to clarify the involvement of the transport protein in the human pharmacokinetics of montelukast. The cell line was validated using E_2_17βG as a standard OATP1B1 substrate since *K*_m_ and *V*_max_ values of 4.1 µM and 697 pmol/mg/min were in line with previously published data [9]. As with the OATP2B1 cell incubations, it was not possible to differentiate between the OATP1B1 and mock cells in terms of montelukast cellular uptake. Consequently, no concentration-dependent uptake experiments were performed, since the results indicate high passive transport and no detectable OATP involvement. Substantiating data was also produced indicating that active transport is at best a minor permeability component for the uptake of montelukast into primary human hepatocytes: Rifampicin was used at a concentration of 10 µM in order to facilitate approximately 80% inhibition of OATP1B1 and OATP1B3 activity [8] but no significant inhibition on the assayed initial montelukast uptake rate was observed. In contrast, the initial E_1_3S uptake rate was reduced by 51%. The back-extrapolated *Y*-intercept of the plots represents instantaneous non-specific drug binding which was understandably higher for montelukast than E_1_3S due to the higher lipophilicity. Taking into account this intercept, the uptake rate between 15 and 60 s appears a good measure of the true initial rate (Figure 4). It is interesting to note that at the 1 min time point, the E_1_3S intracellular concentration was less than 70% of the concentration achieved by 3 min, whereas montelukast uptake was complete after 1 min. This more rapid cellular uptake may be more suggestive of a drug concentration-driven passive process. Nevertheless, the total amount of montelukast entering the cell was reduced in the presence of rifampicin and it cannot be ruled out that the active process plays a minor part in the overall hepatic permeability of the drug. Since 10 µM rifampicin should cause only minimal inhibition of OATP2B1 activity, 4 µM erlotinib was used to selectively inhibit this drug transporter [8]. Erlotinib had no impact on the uptake of either compound, which most likely indicates a low OATP2B1 activity in the hepatocytes used, since E_1_3S is an OATP2B1 substrate.

In summary, it appears unlikely that OATPs are involved in the human absorption of montelukast or distribution into hepatocytes.

## Supplementary Files

Supplementary File 1
