# Supplementary material for: Montelukast Disposition: No Indication of Transporter-Mediated Uptake in OATP2B1 and OATP1B1 Expressing HEK293 Cells
Source: Pharmaceutics. 2015 Dec 15;7(4):554–64. doi: 10.3390/pharmaceutics7040554 (PMC4695834; doi:10.3390/pharmaceutics7040554)
Supplement: Supplementary File 1 [file pharmaceutics-07-00554-s001.pdf]

Supplementary Materials

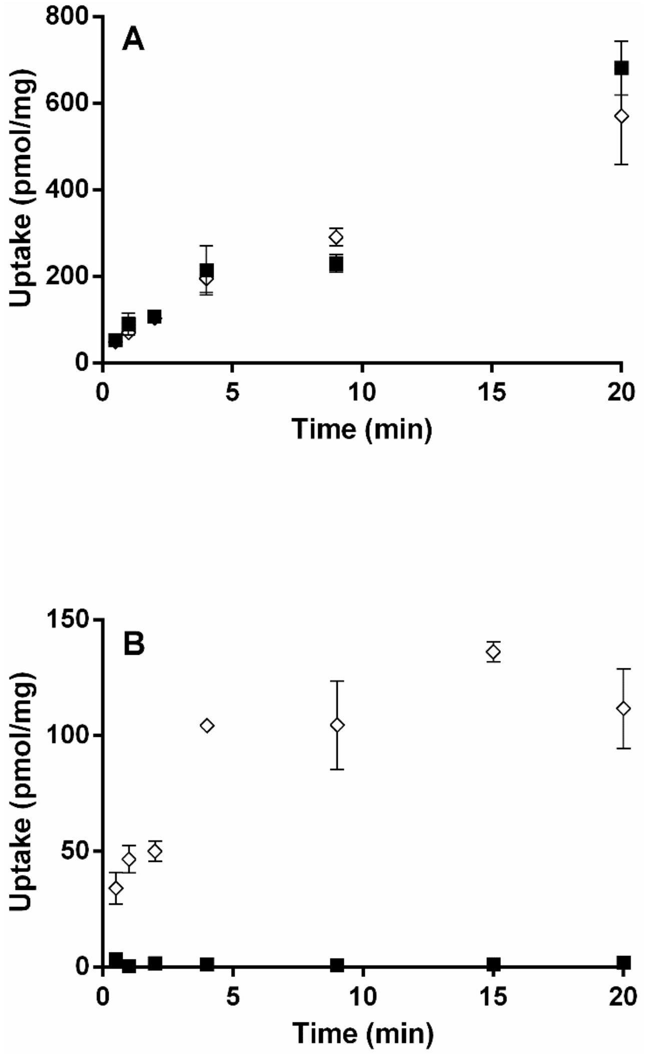

Figure S1. Time-dependent uptake of Montelukast (1 μM) and E_13S (0.5 μM) in HEK-OATP2B1 and HEK-mock at pH 6.5. Results are given as mean ± S.D., *n* = 3.

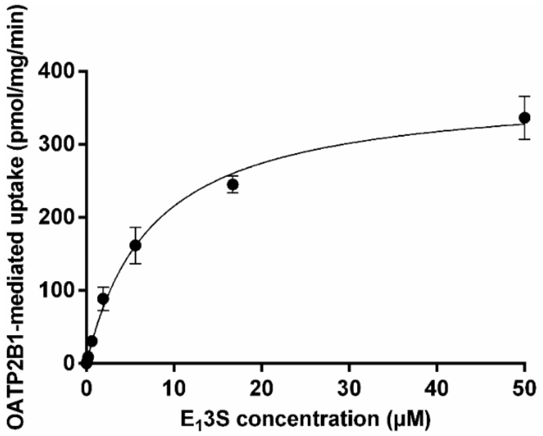

Figure S2. Concentration-dependent uptake of E_13S in HEK-OATP2B1 and HEK-mock at pH 6.5. OATP2B1-mediated uptake, calculated by subtracting the average uptake in HEK-mock from the average uptake in HEK-OATP2B1, was fitted to a Michaelis–Menten equation for K_m and V_{max} determination.

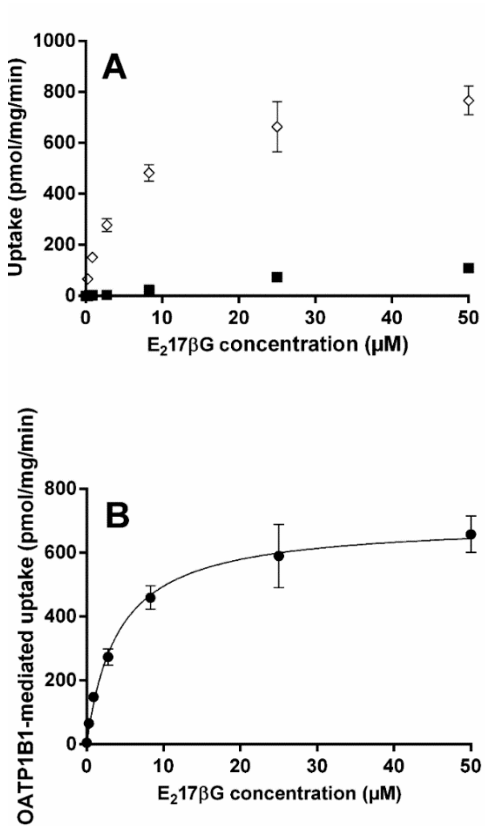

Figure S3. (**A**) Concentration-dependent uptake of $E_217\beta G$ in HEK-OATP1B1 (◊) and HEK-mock (■) cells. Results are given as mean ± S.D., *n* = 3. (**B**) OATP1B1-mediated uptake at pH 7.4, plotted against concentration of $E_217\beta G$. OATP1B1-mediated uptake, calculated by subtracting the average uptake in HEK-mock from the average uptake in HEK-OATP1B1, was fitted to a Michaelis–Menten equation for K_m and V_{max} determination.

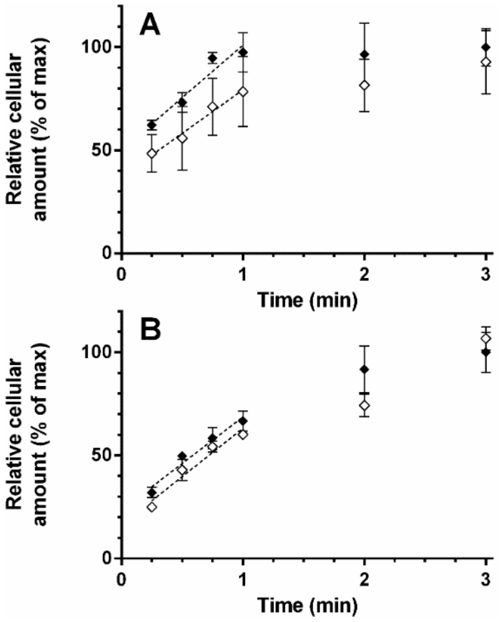

Figure S4. Time-dependent uptake of (**A**) montelukast and (**B**) E_13S into human hepatocytes. Incubations were performed with substrate alone (♦) and in presence of 4 µM erlotinib (◊). Relative initial uptake rates were assessed from linear fit to data obtained from 15 to 60 s (dashed). Results are given as mean ± S.D., *n* = 3.
